# Synaptic protein and pan-neuronal gene expression and their regulation by Dicer-dependent mechanisms differ between neurons and neuroendocrine cells

**DOI:** 10.1186/1749-8104-8-16

**Published:** 2013-08-20

**Authors:** Jutta Stubbusch, Priyanka Narasimhan, Katrin Huber, Klaus Unsicker, Hermann Rohrer, Uwe Ernsberger

**Affiliations:** 1 Max Planck Institute for Brain Research, Deutschordenstrasse 46 D-60528, Frankfurt, Germany; 2 Department of Molecular Embryology, Institute of Anatomy and Cell Biology, University of Freiburg, Albertstrasse 17, D-79104, Freiburg, Germany

**Keywords:** Sympathoadrenal, Synaptic protein, Pan-neuronal, Synaptotagmin, Neurofilament, Dicer 1

## Abstract

**Background:**

Neurons in sympathetic ganglia and neuroendocrine cells in the adrenal medulla share not only their embryonic origin from sympathoadrenal precursors in the neural crest but also a range of functional features. These include the capacity for noradrenaline biosynthesis, vesicular storage and regulated release. Yet the regulation of neuronal properties in early neuroendocrine differentiation is a matter of debate and the developmental expression of the vesicle fusion machinery, which includes components found in both neurons and neuroendocrine cells, is not resolved.

**Results:**

Analysis of synaptic protein and pan-neuronal marker mRNA expression during mouse development uncovers profound differences between sympathetic neurons and adrenal chromaffin cells, which result in qualitatively similar but quantitatively divergent transcript profiles. In sympathetic neurons embryonic upregulation of synaptic protein mRNA follows early and persistent induction of pan-neuronal marker transcripts. In adrenal chromaffin cells pan-neuronal marker expression occurs only transiently and synaptic protein messages remain at distinctly low levels throughout embryogenesis. Embryonic induction of synaptotagmin I (Syt1) in sympathetic ganglia and postnatal upregulation of synaptotagmin VII (Syt7) in adrenal medulla results in a cell type-specific difference in isoform prevalence. Dicer 1 inactivation in catecholaminergic cells reduces high neuronal synaptic protein mRNA levels but not their neuroendocrine low level expression. Pan-neuronal marker mRNAs are induced in chromaffin cells to yield a more neuron-like transcript pattern, while ultrastructure is not altered.

**Conclusions:**

Our study demonstrates that remarkably different gene regulatory programs govern the expression of synaptic proteins in the neuronal and neuroendocrine branch of the sympathoadrenal system. They result in overlapping but quantitatively divergent transcript profiles. Dicer 1-dependent regulation is required to establish high neuronal mRNA levels for synaptic proteins and to maintain repression of neurofilament messages in neuroendocrine cells.

## Background

A key property of neurons and neuroendocrine cells is the regulated release of chemical messengers to convey information to target cells across microscopic distances for the neurotransmitters at the synaptic cleft or along macroscopic dimensions for humorally distributed hormones. The cellular machinery to achieve the regulated release of these chemical messengers relies in part on the same vesicular, cytoplasmic and plasma membrane proteins [1,2]. Peripheral sympathetic neurons and adrenal chromaffin cells, derived from the same precursor population, the neural crest, provide an excellent model system to analyze the mediators regulating the divergence of neuronal and neuroendocrine cell fates [3].

Early studies of rodent development shaped the idea that both sympathetic neurons and adrenal chromaffin cells originate from the same sympathoadrenal progenitor cell, which already displays some neuronal features such as low molecular weight neurofilament protein (NF-L) [4-8]. While the generation of sympathetic neurons and adrenal chromaffin cells from common precursors has been demonstrated by single cell electroporation of GFP-DNA into the dorsal neural tube and delaminating neural crest cells in vivo [7,9], the molecular specification of this precursor as well as the precise timing and location of divergence between the sympathetic neuronal and adrenal neuroendocrine lineage is unclear. The lack of medium molecular weight neurofilament protein (NF-M) expression during chromaffin cell differentiation in the chick embryo [10] challenges the classical model of the molecular specification of the sympathoadrenal progenitor as a cell expressing neuronal features [4-8]. Moreover, the molecular characteristics of mature chromaffin cells as compared to their neuronal counterparts as well as the regulatory processes governing their developmental acquisition are poorly understood.

In sympathetic ganglia, neuronal differentiation mediated by aorta-derived bone morphogenetic proteins involves early induction of pan-neuronal properties such as neurofilaments and stathmin-2, also known as superior cervical ganglia, neural specific 10 (SCG10) [11-13]. Studies in chick embryos show that synaptic protein expression, as exemplified by synaptotagmin I (Syt1), follow with some delay and a slow increase in mRNA accumulation [14]. This pattern of sequential expression of mRNAs for pan-neuronal and synaptic proteins with different time courses of mRNA accumulation is shared by other peripheral and central neuron populations [15], and provokes the question as to the contribution of transcriptional and post-transcriptional regulation.

In the adrenal medulla, the regulation of pan-neuronal and synaptic proteins is less well defined. The early NF-L expression observed in rodents is lost with ongoing embryonic development [16,17] suggesting a general downregulation of pan-neuronal markers in differentiating chromaffin cells. In chick embryos, however, the absence of mRNA for NF-M serves as an indicator for prospective chromaffin cells from the onset of adrenal tissue formation and argues against an early expression of a large set of neuronal markers in adrenal chromaffin precursors [10]. In addition, Syt1 mRNA levels remain strikingly low throughout embryonic development. Occasionally found spots of cells with elevated NF-M and Syt1 mRNA levels preferentially at the outer margin of adrenal tissue [10] are attributed to a small number of neurons observed in adrenal tissue [18].

Electrophysiological studies in mutant mice demonstrate that Syt1, the key calcium sensor for fast evoked transmitter release in neurons [19], mediates the fast component of catecholamine release in adrenal chromaffin cells of late embryonic and neonatal animals [20,21]. On the other hand, synaptotagmin VII (Syt7), which appears not to be required for neuronal transmitter release [22], mediates the large slow component of catecholamine release in chromaffin cells [21]. These findings prompt the question of whether expression of the components of the vesicle fusion machinery is differentially controlled in neurons and neuroendocrine cells, and how the mature situation is established during development.

Regulation by various transcription factors has been demonstrated to markedly affect catecholaminergic differentiation in sympathoadrenal cells [3,23,24], but has failed to provide critical insight into the divergence of the neuronal and neuroendocrine lineages recommending the study of other candidate regulator classes. Post-transcriptional control and in particular microRNAs have been established as potent regulators of neuronal gene expression patterns [25-28]. Importantly, the reprogramming of fibroblasts and subsequent neuronal differentiation by selected microRNAs [29] demonstrates the powerful role this class of regulators may exert during cell type specification. Interference with microRNA maturation by conditional inactivation of Dicer 1 in neural crest cells affects the expression of subset and transmitter phenotype-specific properties in sympathetic neurons [30,31], prompting the question for their involvement in the neuronal-neuroendocrine diversification.

To address these issues, we analyzed the expression pattern of genes involved in synaptic function during embryonic and postnatal mouse development, and the effects of Dicer 1-mediated regulation. Comparative analysis of Syt1 and Syt7 with Snap25, which is crucially involved in catecholamine release in neuronal and chromaffin cells [32,33], and Ras-related protein Rab-3A (Rab3a), an established model for transcriptional regulation of a gene coding for synaptic proteins [34], demonstrates profound differences in the transcript patterns for synaptic proteins between neuronal and neuroendocrine cells. Comparison to pan-neuronal genes supports the concept of a gene expression program conserved between different neuronal classes, and its divergence between neurons and chromaffin cells during embryonic and postnatal development. The search for regulators involved in this lineage dichotomy shows the microRNA-synthesizing RNase Dicer 1 to be involved in suppression of pan-neuronal gene expression in neuroendocrine cells and maintenance of message levels for synaptic proteins in neurons.

## Results

### Qualitatively similar but quantitatively different expression profiles for synaptic protein mRNAs distinguish mature adrenal chromaffin cells and sympathetic neurons

Synaptic proteins required for neuronal transmitter release are also expressed in neuroendocrine cells and required for hormone secretion [1,2]. However, protein levels differ, as observed by immunohistochemistry in rat sympathetic neurons and adrenal neuroendocrine cells [35], and Syt1 mRNA levels in chromaffin cells are low compared to neuronal cells including the sparse neurons found in the adrenal medulla [36]. While Syt1, which is essential for regulated transmitter release in neurons [19], contributes only a small fast component to chromaffin cell vesicle fusion, Syt7 mediates the larger slow component of catecholamine release in adrenal chromaffin cells [21]. To analyze how this is reflected in the gene expression pattern, sympathetic ganglia and adrenal glands were analyzed by in situ hybridization (ISH) for several synaptic protein mRNAs in 3-week-old to 3-month-old mice.

**Figure 1:**
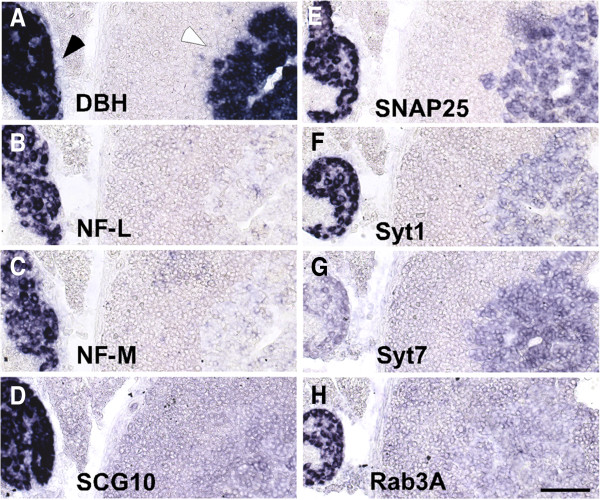
**Inverse mRNA ratios for Syt1 and Syt7 are found in P21 adrenal chromaffin cells and sympathetic neurons. (A)** ISH for DBH mRNA marks noradrenergic sympathetic neurons in a prevertebral ganglion (black arrowhead) and chromaffin cells in the adrenal medulla (white arrowhead) at P21. Sympathetic neurons display strong signals for **(B)** NF-L, **(C)** NF-M and **(D)** SCG10 mRNAs, which are barely detectable in adrenal chromaffin cells. **(E)** Snap25 mRNA is detected at high levels in the majority of neurons and at intermediate levels in the majority of neuroendocrine cells, while **(F)** Syt1 mRNA is strongly expressed in neurons but only weakly in chromaffin cells. **(G)** Low expression levels of Syt7 mRNA in sympathetic ganglia contrast with medium to high signal strength in adrenal medulla. **(H)** Rab3a mRNA is detected at high levels in neurons but not above background in chromaffin cells. Scale bar: 100 μm.

Adjacent to the adrenal gland, the suprarenal ganglion, a sympathetic ganglion attached to the surface of the adrenal cortex and connected with the celiac/superior mesenteric ganglion complex [37,38], is detected by ISH for dopamine β-hydroxylase (DBH) allowing direct comparison of adult neuronal and endocrine gene expression patterns. Striking differences in mRNA levels are observed not only for pan-neuronal markers such as NF-M, which demarcate neuronal tissue, but also for mRNAs coding for synaptic proteins (Figure 1). At postnatal day 21 (P21), a detailed analysis was performed when an expression pattern is observed in adrenal medulla that does not change grossly until postnatal day 90 (P90). Of importance, Syt7 mRNA levels in adrenal medulla exceed those in sympathetic ganglia, which correlate with its respective role in catecholamine secretion [21]. In contrast, Syt1 mRNA signals are considerably higher in neuronal than neuroendocrine tissue. Also Snap25 mRNA is detectable at higher levels in ganglia. Interestingly, Rab3a mRNA signals are easily detected in mature neuronal but not chromaffin tissue. Although Rab3a is the most abundant Rab3 isoform in adrenal tissue as indicated by western blot [39], neuroendocrine Rab3a message levels appear dramatically lower than in neurons and are below the threshold of the detection method. As expected, the pan-neuronal markers NF-M, NF-L and SCG10 are highly expressed in sympathetic neurons but not adrenal chromaffin cells.

Taken together, the results demonstrate a profound difference in synaptic protein and pan-neuronal gene expression patterns between mature neuronal and neuroendocrine tissue. In particular, the differential abundance of mRNAs for isoforms Syt1 and Syt7 corresponds to their diverging roles in neuronal and neuroendocrine vesicle fusion. Axonal transport of mRNAs to synaptic sites may add to the ISH signals observed. However, since preganglionic boutons contribute only approximately 1% to the area of sympathetic ganglia [40] and mRNA in preganglionic axons traversing the ventral root is below detection threshold for the ISH protocol (our own observations), the mRNA signals can be largely attributed to sympathetic neuron and adrenal chromaffin cell bodies. A possible axonal transport in the former cell type, however, may result in an underestimation of the difference in mRNA levels of neuronal and neuroendocrine cells.

### Expression of genes coding for synaptic and pan-neuronal proteins marks an early segregation of neuronal and neuroendocrine lineages

The common catecholaminergic phenotype in combination with the divergent neuronal or neuroendocrine character of sympathetic neurons and adrenal chromaffin cells, respectively, makes the developmental analysis of early lineage diversification particularly interesting. The traditional view of an initially neuronal specification of the entire progenitor pool, as concluded from a transient prevalence of NF-L expressing cells in embryonic rat adrenal medulla [16], conflicts with the almost complete lack of NF-M and Syt1 mRNAs in differentiating adrenal chromaffin cells in the chick embryo [10]. To analyze whether this divergence is evidence for differences between rodent and chick development, we studied the expression patterns of mRNAs for synaptic proteins and pan-neuronal markers at E12.5 in the mouse embryo when adrenal anlagen form.

**Figure 2:**
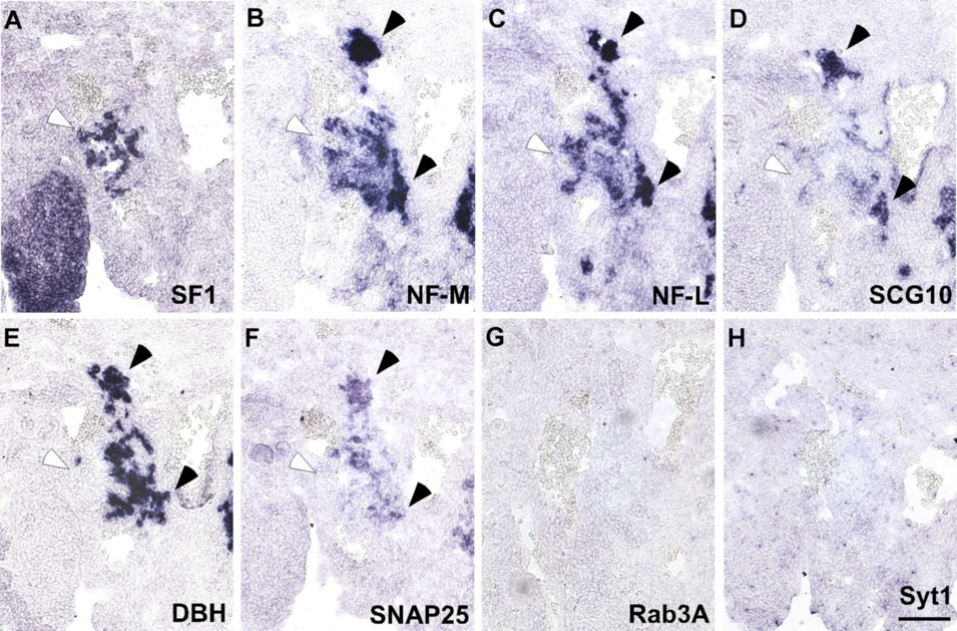
**Differential expression of mRNAs coding for pan-neuronal and synaptic proteins in anlagen of adrenal gland and sympathetic ganglia at E12.5. (A)** In consecutive sections from E12.5 mouse embryos, the position of the adrenal anlagen is marked by ISH for Nr5a1, also known as steroidogenic factor (SF1), expressed in cells destined to form prospective adrenal cortex (white arrowhead). Ganglion primordia (black arrowheads) show strong signals for **(B)** NF-M and **(C)** NF-L, which are also observed, albeit often at lower levels in adrenal anlagen. **(D)** SCG10 signals are barely detected in adrenal anlagen but are strong in ganglion primordia. **(E)** Catecholaminergic cells of the sympathoadrenal lineage are shown by expression of DBH in the adrenal anlage adjacent to Nr5a1-positive cells and in primordia of sympathetic ganglia. While **(F)** Snap25 is expressed in ganglion and adrenal anlagen, **(G)** Rab3a and **(H)** Syt1 signals are not above background. As all labeled cell populations may still be migratory active, those contributing to adrenal medulla and the different sympathetic ganglia, including the paraaortic, suprarenal and celiac-superior mesenteric ganglion complex, cannot safely be attributed at this stage. The distribution of the Nr5a1-positive cells roughly marks the adrenal anlage. Scale bar: 100 μm.

To locate the sites of adrenal gland formation, mRNA for the nuclear receptor subfamily 5, group A, member 1 (Nr5a1), also known as steroidogenic factor 1 (SF1), expressed in prospective adrenal cortex cells, was compared to DBH in differentiating sympathoadrenal cells (Figure 2). DBH and Nr5a1 mRNA-expressing cells are not yet segregated at E12.5. In the Nr5a1-demarcated area, NF-L is detected as reported previously [41]. NF-M mRNA is also abundantly expressed at E12.5 with clusters of weakly positive cells found predominantly in the adrenal anlagen. Aggregates of cells expressing NF-M and NF-L at particularly high levels similar to paravertebral sympathetic ganglia are found dorsally to the Nr5a1-positive domain in the region typically attributed to primary sympathetic ganglia and ventromedially likely contributing to the suprarenal ganglion [17,41]. In these regions SCG10 mRNA is also highly expressed, further supporting the neuronal character of these aggregates. In contrast, low expression if any at all is detected in the Nr5a1-positive region for this marker. Different from SCG10, Snap25 mRNA signals are detected in adrenal and ganglionic regions, while Syt1 as well as Rab3a mRNA signals are not above background.

In neighboring, more cranially located paravertebral sympathetic ganglia, mRNAs for NF-M and NF-L are highly expressed, SCG10 and Snap25 occur at intermediate levels, while Syt1 and Rab3a mRNAs appear low or undetectable, respectively (data not shown). The difference in Syt1 expression in the neuronal structures at this stage may be explained by the rostrocaudal differentiation gradient observed during sympathetic neurogenesis.

These results demonstrate an early induction of Snap25, NF-M and NF-L mRNAs, common to adrenal anlagen and sympathetic ganglia. However, differences in SCG10 expression and neurofilament mRNA levels indicate a very early divergence in the gene regulatory program between the neuronal and neuroendocrine lineages.

### mRNAs for synaptic proteins become enriched in embryonic sympathetic neurons but not adrenal chromaffin cells

At the end of the second embryonic week, segregation of the cells contributing to adrenal medulla, adrenal cortex and suprarenal ganglion is largely complete. During the third week of embryogenesis adrenal chromaffin cells appear ultrastructurally differentiated and display the characteristic catecholamine storage in large dense-core vesicles (LDCVs) [42]. Regulated fusion of these vesicles and hormone release is observed in chromaffin cells of E18 mouse embryos [21,33,39]. In the alternative branch of sympathoadrenal development leading to sympathetic neurons, cellular differentiation including neurite outgrowth occurs during the second week of mouse embryogenesis [43,44] and target innervation commences in the third week [45-49]. To compare expression of genes coding for synaptic proteins and pan-neuronal markers during this period of intense morphological and physiological differentiation, we chose adrenal gland and sympathetic ganglia from E16 and P0 mice.

**Figure 3:**
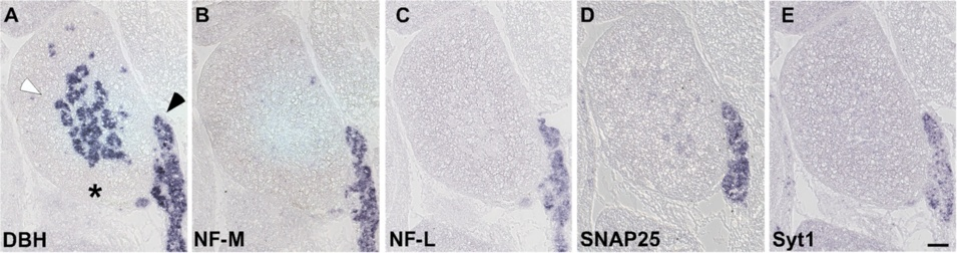
**Absence of mRNAs for pan-neuronal and synaptic proteins in adrenal gland as compared to sympathetic ganglia at advanced embryonic stage (E16.5). (A)** At E16.5, adrenal medulla (white arrowhead) and cortex (asterisk) as well as suprarenal ganglion (black arrowhead) are largely segregated as shown by ISH for DBH. While ganglionic neurons display strong mRNA signals for **(B)** NF-M, **(C)** NF-L, **(D)** Snap25 and **(E)** Syt1, adrenal chromaffin cells lack these markers except for weak **(D)** Snap25 signals. Scale bar: 100 μm.

In mouse embryos at E16.5, when cells of the adrenal medulla have already acquired chromaffin cell properties including LDCVs [42], Syt1 mRNA is not detected by ISH in adrenal tissue (Figure 3), while the Snap25 mRNA signal appears weak but distinctly above background in at least some adrenal medulla cells. mRNAs for NF-M and NF-L are not observed except for few cells likely corresponding to scattered neurons in the adrenal medulla. In contrast, prevertebral neuron clusters immediately adjacent to the adrenal gland show strong signals for SCG10 (not shown), NF-M, NF-L and Snap25 (Figure 3). At the same time, mRNA for Syt1 is neuronally expressed, while message for the synaptic small GTPase Rab3a is not yet detectable (data not shown).

The pattern of mRNA expression is similar in cells of the adrenal medulla in newborn mice (see below). Snap25 mRNA signals are observed, albeit at low levels compared to neurons of adjacent prevertebral ganglia. Syt1 mRNA gives very weak signals and Syt7 message signals appear barely above background in adrenal chromaffin cells. Rab3a message signals remain below threshold for detection with the ISH protocol employed. In contrast, in prevertebral neuron clusters of newborn mice, the mRNAs for the synaptic proteins are distinctly expressed with Snap25 and Syt1 detectable at high levels, while Rab3a and Syt7 mRNAs give only modest signals. Pan-neuronal mRNAs for NF-L, NF-M and SCG10 are highly expressed in prevertebral neuron clusters but not in adrenal chromaffin cells.

These data demonstrate that the gene expression programs for synaptic proteins in sympathetic neurons and adrenal chromaffin cells differ during embryonic development when catecholamine release by regulated vesicle fusion becomes established. Remarkably, Rab3a mRNA remains below ISH detection limit in chromaffin cells even when the protein function is detectable with physiological techniques [21,39].

### Dicer inactivation induces mRNAs for NF-M and NF-L but reduces Syt1 and Snap25 mRNAs in sympathetic neurons

Conserved features of gene expression during neuronal development are the delayed induction of Syt1 as compared to neurofilament expression during early differentiation stages [15] as well as the slow increase in synaptic protein mRNA levels during advanced stages of neuronal development. To search for evidence of microRNA-mediated regulation in these processes, Dicer 1 was conditionally inactivated by DBH promoter-driven Cre recombinase [50-52].

**Figure 4:**
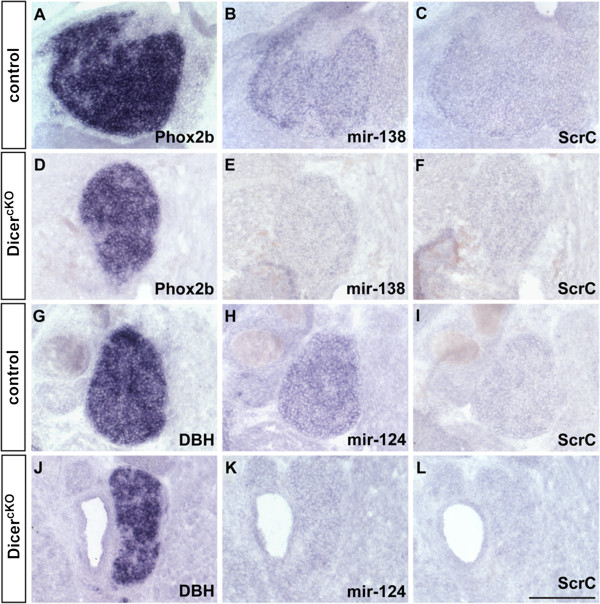
**Conditional Dicer inactivation suppresses miR-124 and miR-138 signal in sympathetic neurons.** In E16.5 mouse embryo, **(A**, **D)** Phox2b and **(G**, **J)** DBH ISH signal marks the position of the SCG in **(A**, **G)** control and **(D**, **J)** a mutant animal with homozygous inactivation of floxed Dicer by DBH promoter-driven Cre recombinase (Dicer^cKO^). **(B**, **E)** miR-138 and **(H**, **K)** miR-124 are detected by LNA-ISH in **(B**, **H)** control sympathetic neurons and reduced in **(E**, **K)** mutant ganglia. LNA-ISH with **(C**, **F**, **I**, **L**) scrambled sequence probe shows specificity of ISH signals. Scale bar: 100 μm.

**Figure 5:**
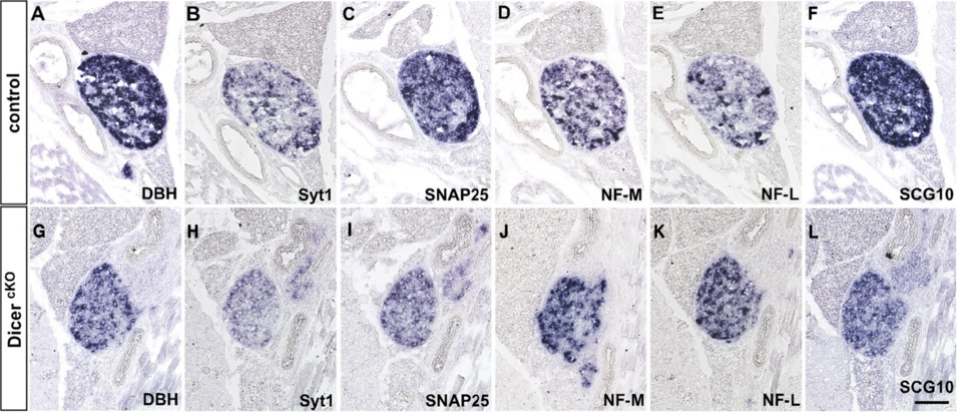
**Enhanced neurofilament and reduced synaptic protein mRNA levels in the SCG of newborn mice with conditional Dicer 1 inactivation.** In newborn mice, DBH ISH signal marks the position of the SCG in **(A)** control and **(G)** a mutant animal with homozygous inactivation of floxed Dicer 1 by DBH promoter-driven Cre recombinase (Dicer^cKO^). **(B**, **H)** Syt1 and **(C**, **I)** Snap25 ISH signals are reduced in mutant animals, while **(D**, **J)** NF-M and **(E**, **K)** NF-L mRNA signals are elevated. **(F**, **L)** Different from the neurofilaments, SCG10 mRNA signal is reduced. Scale bar: 100 μm.

At E16.5, 6 days after the onset of endogenous DBH expression in differentiating sympathetic neurons, microRNAs miR-124 and miR-138 can be detected by locked nucleic acid (LNA)-ISH in sympathetic ganglia of control but not of mutant embryos (Figure 4). At this stage no gross alterations in mRNA levels for synaptic proteins, in particular Rab3a, became apparent in sympathetic ganglia (data not shown) arguing against the possibility that Dicer 1-dependent regulation is essential for the delay in synaptic protein expression during this period of neuronal differentiation. However, in newborn superior cervical ganglia (SCG), signals for Syt1 and Snap25 mRNAs are reduced in mutant as compared to control animals (Figure 5). In contrast, NF-M and NF-L mRNA signals are increased in mutant ganglia. Different from neurofilaments, mRNA levels for SCG10 are reduced in mutant animals.

Since Dicer 1 inactivation in neural crest cells results in reduction of sympathetic ganglion size due to cell loss [30,31], we analyzed whether altered mRNA expression patterns may be due to selective loss of specific neuron populations. Control ganglia in newborn animals show a small subset of neurons with strong neurofilament expression, whereas the majority of cells express NF-M or NF-L mRNA at intermediate or low levels, respectively (Figure 5). In contrast, mutant animals display more neurons with neurofilament mRNA signals of strong or intermediate strength. Moreover, in newborn mutant mice, SCG volume is reduced by approximately 70% to 0.014 ± 0.002 mm^3^ (n = 6) as compared to 0.049 ± 0.005 mm^3^ (n = 6) in control, largely due to an 86% (n = 5) reduction in the size of the DBH-positive cell population (Stubbusch et al., in preparation). Quantifying the proportion of the SCG with NF-L mRNA expression levels of different strength (see Methods), shows that strong and intermediate signal amounts to 9.1 ± 2.9% and 17.9 ± 1.9% (n = 3), respectively, of DBH-positive area in mutant ganglia as compared to 5.2 ± 1.7% and 8.4 ± 1.7% (n = 3) in control. The moderate increase in the relative proportion of the two populations of neurons with enhanced NF-L ISH signals occurs in parallel with a decrease in the volume of the residual mutant ganglion occupied by cells displaying strong, intermediate as well as weak NF-L mRNA signals. This loss in absolute population sizes amounts to 72% for cells with strong NF-L signal levels, 67% for cells with intermediate NF-L signal levels and 83% for cells with weak NF-L signal levels. Similar numbers are obtained for NF-M. The two- to threefold increase in the proportion of neurons with elevated neurofilament ISH signals in contrast to the major loss of ganglion size does not agree with a simple loss of cells with low neurofilament message levels to explain the increased mRNA signals in mutant ganglia. Instead, an increase of neurofilament mRNA levels across neuronal subpopulations despite ongoing cell loss best explains the observations.

These data indicate that mRNA levels for synaptic proteins are increased and those for NF-M and NF-L are reduced by Dicer 1 activity in parallel to maintenance of sympathetic neurons during advanced neuronal differentiation.

### Dicer inactivation induces expression of pan-neuronal genes in adrenal chromaffin cells

The mechanisms underlying transient expression of pan-neuronal properties in differentiating neuroendocrine cells as well as the distinct temporal expression profiles of genes coding for synaptic proteins are currently not understood. The increase in neurofilament mRNA levels observed in sympathetic neurons prompts the question whether Dicer inactivation derepresses neurofilament expression in adrenal chromaffin cells.

**Figure 6:**
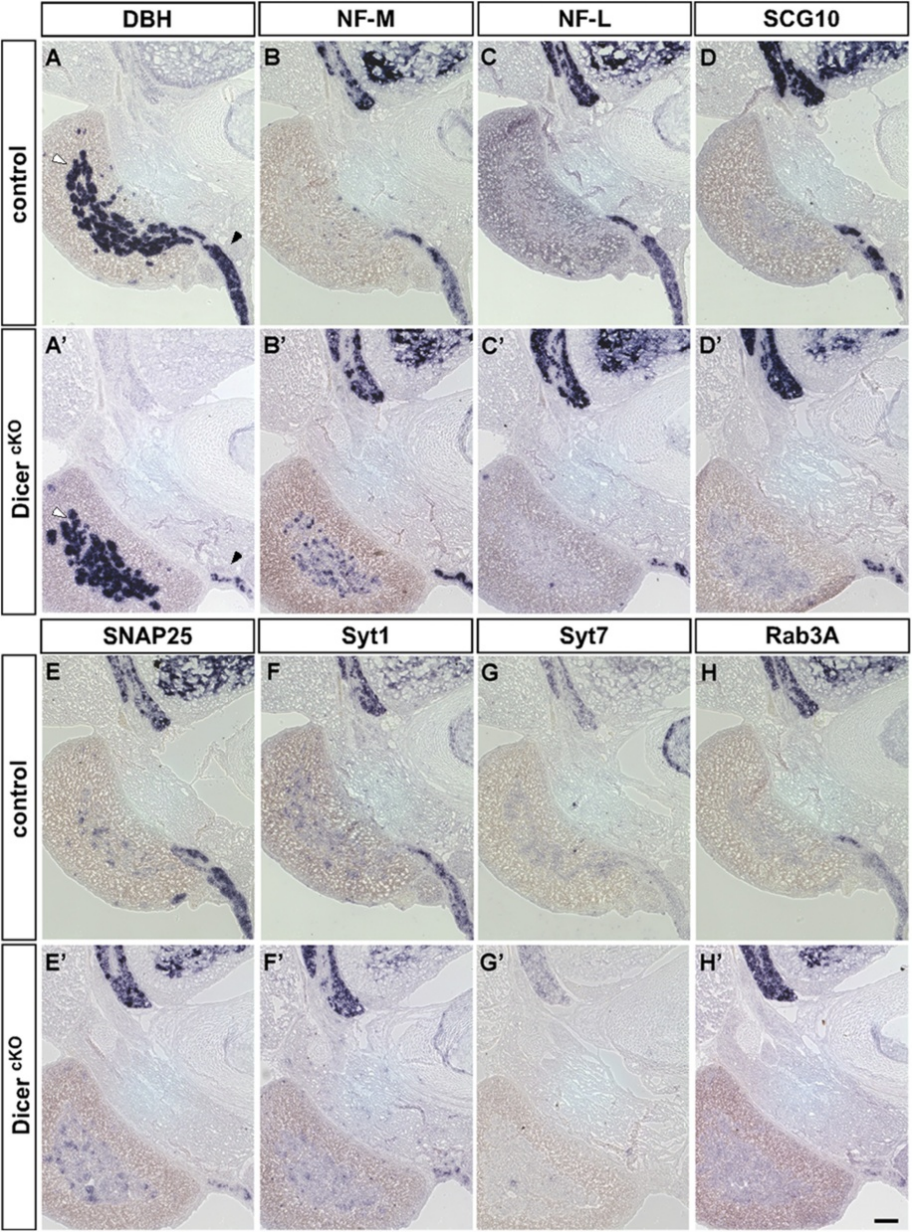
**NF-M but not other pan-neuronal and synaptic protein mRNAs is derepressed in the adrenal medulla of newborn Dicer mutant mice. (A**,**B**,**C**,**D**,**E**,**F**,**G**,**H)** ISH on transverse trunk sections from a newborn control mouse and **(A**’,**B**’,**C**’,**D**’,**E**’,**F**’,**G**’,**H**’**)** an animal with homozygous inactivation of floxed Dicer by DBH promoter-driven Cre recombinase. **(A**, **A**’**)** DBH ISH signal marks the position of the adrenal medulla (white arrowhead) and a prevertebral neuron cluster (black arrowhead). The neurons of the sympathetic ganglion display strong mRNA signals for **(B)** NF-M, **(C)** NF-L, **(D)** SCG10, **(E)** Snap25 and **(F)** Syt1, similar to neurons in the dorsal root ganglion and the ventral spinal cord. Abundant NF-M mRNA signal is also detected in adrenal medulla of **(B**’**)** mutant animals but not in **(B)** control. However, **(C**’**)** NF-L and **(D**’**)** SCG10 mRNAs do not appear upregulated in adrenal medulla. **(E)** Snap25 and **(F)** Syt1 mRNA signals are strong in neurons, and appear low in adrenal medulla of control animals. In homozygous mutants, **(E**’**)** Snap25 and **(F**’**)** Syt1 appear unaffected in adrenal medulla but reduced in prevertebral neuron clusters. **(G)** Syt7 mRNA signals are very low in control, and **(G**’**)** undetectable in mutant adrenal medulla and sympathetic neuron clusters. **(H**, **H**’**)** Rab3a mRNA signals are high in the dorsal root ganglion and the ventral spinal cord, and weakly detected in control and mutant sympathetic neuron clusters but not in adrenal medulla. Adjacent sections were used for ISH with the probes indicated, and experiments were performed to compare three mutant and three control animals for each individual probe. The panels from a representative animal are shown in this figure. Scale bar: 100 μm.

**Figure 7:**
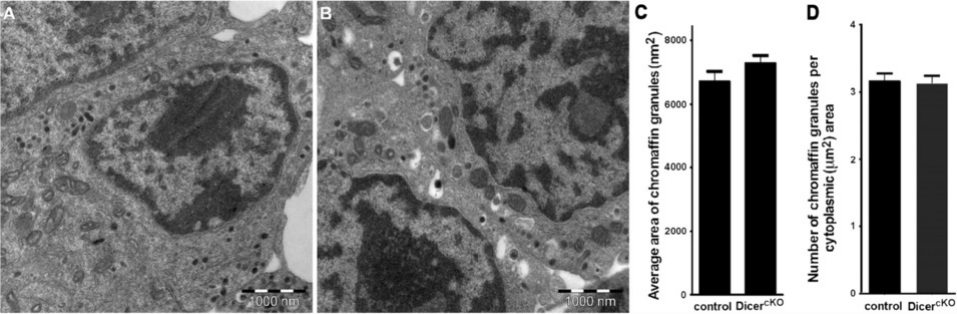
**Conditional Dicer inactivation does not alter the ultrastructure of adrenal chromaffin cells.** Electron micrographs show P0 adrenal chromaffin cells from **(A)** control and **(B)** mutant (Dicer^cKO^) mice. Quantitative analysis fails to reveal significant differences in **(C)** chromaffin granule size and **(D)** number of chromaffin granules per unit cytoplasmic area between control and mutant animals. Scale bar: 1,000 nm.

In newborn mice from matings of animals carrying the DBH promoter/Cre transgene and the floxed *Dicer 1* gene, adrenal medulla is not reduced in size, and can be directly compared with adjacent neuronal aggregates attributed to the suprarenal and celiac ganglia (Figure 6). Whereas adrenal chromaffin cells from control animals display no ISH signal for NF-M, NF-L or SCG10 mRNAs, NF-M but not NF-L or SCG10 signal is markedly upregulated in the adrenal medulla of homozygous mutants. Interestingly, the low Syt1 and Snap25 mRNA signals detected in control adrenal tissue are not reduced in mutants. Electron microscopic analysis shows no alteration in size and density of catecholamine storage vesicles (Figure 7) indicating that the neuroendocrine phenotype of the chromaffin cells is maintained.

Similar to newborn animals, at postnatal day 6 (P6), mice carrying the homozygous Dicer 1 inactivation show enhanced NF-M mRNA levels in the adrenal medulla, albeit only in a subset of cells and mostly at levels well below those found in neurons (data not shown). Cells with NF-L mRNA signals appear occasionally in the adrenal medulla of homozygous mutants but constitute only a small minority. SCG10 mRNA signals in adrenal medulla of these animals may be slightly higher than in controls but remain far below those in neurons. No induction of Syt1 mRNA is observed after Dicer 1 inactivation. Thus, NF-M induction in chromaffin cells upon Dicer 1 inactivation is not indicative of a comprehensive neuronal transdifferentiation.

**Figure 8:**
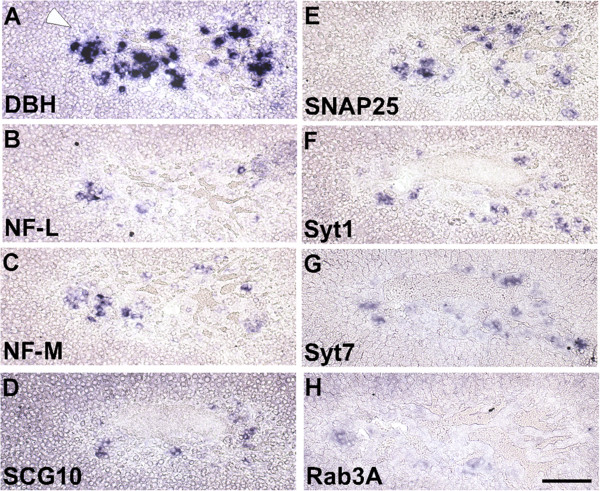
**Mixed neuronal/neuroendocrine mRNA expression pattern in P21 adrenal medulla after conditional Dicer inactivation. (A)** Adrenal medulla in a mouse with conditional Dicer 1 ablation is shown by DBH ISH (white arrowhead). **(B)** NF-L and **(C)** NF-M mRNA expression is detected in a substantial fraction of cells in the remaining adrenal medulla, and **(D)** SCG10 signals are well above background. Prominent signals are observed for **(E)** Snap25, **(F)** Syt1 and **(G)** Syt7 mRNAs, and **(H)** Rab3a-positive cells are occasionally detected. Adjacent sections were used for ISH with the probes indicated, and experiments were performed to compare three mutant and three control animals for each individual probe. The panels from a representative homozygous mutant animal shown in this figure can be directly compared with those in Figure 2 from a representative control animal. Scale bar: 100 μm. DBH, dopamine β-hydroxylase; ISH, in situ hybridization; NF-L, low molecular weight neurofilament protein; NF-M, medium molecular weight neurofilament protein; P21, postnatal day 21; SCG10, superior cervical ganglia, neural specific 10; Snap25, synaptosomal-associated protein 25; Syt1, synaptotagmin I; Syt7, synaptotagmin VII.

At postnatal day 21 (P21), the size of the adrenal medulla in Dicer mutant animals is considerably smaller than in control and adrenal gland-associated ganglia are missing in mutant mice. In addition to NF-M, NF-L and SCG10 mRNAs are abundantly expressed in a subset of cells (Figure 8). Syt1, Syt7 and Snap25 mRNAs are retained in a large fraction of the remaining medulla cells in mutant tissue. The levels are, however, not shifted to those seen in prevertebral neurons of control animals at this stage (Figure 1). Different from control, weak Rab3a mRNA signals are observed in some cells of mutant adrenal medulla, which again do not show intensity levels of control neurons.

The data demonstrate that conditional Dicer 1 inactivation in catecholaminergic cells of the adrenal medulla derepresses neurofilament expression similar to sympathetic neurons. Different from the effect in neurons, however, mRNA levels for synaptic proteins are not reduced. Remaining cells express both Syt1 and Syt7 at moderate levels, thus sharing neuronal and neuroendocrine properties.

## Discussion

The assembly of the vesicle fusion machinery during embryonic differentiation of neurons and neuroendocrine cells is largely unresolved, and the regulatory mechanisms governing expression of the genes coding for synaptic proteins as well as the coordination with other cell type-specific features are poorly defined. Here, analyzing a selected set of mRNAs for synaptic and pan-neuronal proteins in mouse sympathoadrenal cells, we show a profound difference in their expression between neurons and neuroendocrine cells. In neurons, both gene sets are expressed at early embryonic stages preceding the period of target innervation as also observed in other vertebrate neuron populations. In adrenal chromaffin cells, some pan-neuronal genes are transiently expressed embryonically, while mRNA accumulation for synaptic proteins is delayed and occurs largely postnatally. Dicer 1-mediated increase in mRNA levels for synaptic proteins in developing neurons and Dicer 1-regulated repression of pan-neuronal markers in differentiating chromaffin cells contribute to the divergent gene expression profiles in sympathetic neural and adrenal neuroendocrine tissue.

### Differences in synaptic protein mRNA levels and sensitivity to Dicer 1 inactivation imply different gene expression programs in neurons and neuroendocrine cells

Catecholamine release from chromaffin cells shows a major slow component [20] that is carried by a specific molecular mechanism involving Syt7 [21]. Syt1 mediates a fast component of vesicle fusion, which contributes only a small part of catecholamine secretion in these cells [20,21]. This differs from neurons where a rapid Syt1-dependent process may carry the major proportion of regulated transmitter release followed by a minor slow component [19]. In line with these findings we observe different ratios of Syt1 and Syt7 expression in adrenal chromaffin cells and sympathetic neurons with Syt1 mRNA levels enriched in neurons and Syt7 mRNA in neuroendocrine cells.

Low Syt1 message levels are not only observed in mouse adrenal chromaffin cells but also in rat [36] and chick [10] adrenal medulla. Moreover, in adult rat pituitary, Syt1 mRNA is detected at levels approximately an order of magnitude smaller than in neighboring hypothalamic neurons [53] suggesting that low Syt1 mRNA expression is shared by different classes of endocrine cells.

High neuronal and low adrenal Syt1 mRNA levels are already observed during embryogenesis. Similar differences in message levels are also found for Snap25 and Rab3a, albeit at variable degrees. The observation that Dicer 1 inactivation reduces synaptic protein mRNA levels in sympathetic ganglia but not adrenal medulla indicates that regulation differs in neurons and neuroendocrine tissue.

Thus, large quantitative differences in synaptic protein mRNA levels in mature cells, including opposite Syt1:Syt7 mRNA ratios, correlate with physiological differences in vesicle fusion between neuronal and neuroendocrine cells. Profoundly distinct developmental profiles of mRNA accumulation, which differ in sensitivity to Dicer 1 inactivation, suggest that diverging gene regulation programs govern neuronal and neuroendocrine expression of synaptic proteins.

### The role of Dicer 1 and transcription factors for synaptic protein and pan-neuronal marker expression during neuronal development

The molecular mechanisms underlying the initial expression of synaptic proteins during neuronal differentiation are currently not resolved. Expression of the enzymes required for catecholamine biosynthesis in sympathetic neurons, tyrosine hydroxylase (TH) and DBH, is tightly coupled and follows shortly after induction of the transcription factor network regulating their expression [23,54]. Paired-like homeobox 2b (Phox2b), the key player in this network, is able to induce not only TH and DBH but also Syt1 upon ectopic expression [55]. Pan-neuronal markers such as SCG10 and NF-M are also induced [56], indicating that Phox2b is able to trigger a comprehensive neuronal differentiation program entailing generic and subtype-specific features.

The induction of various neuronal features by Phox2b is in agreement with observations from the Phox2b mouse mutant where sympathetic neuron differentiation is blocked at a precursor stage [43]. Since adrenal chromaffin cell differentiation is similarly abrogated before the onset of catecholaminergic development or neurofilament expression [41] this transcription factor is prerequisite for neuronal and neuroendocrine differentiation. Persistence of beta-III tubulin in sympathetic ganglia of mice with conditional Phox2b inactivation in already differentiated neurons [57] suggests that Phox2b initiates an autonomic differentiation program without being required to maintain expression of pan-neuronal features.

However, the sequential upregulation of neuronal markers during sympathetic neuron differentiation is currently not understood in terms of the Phox2b-dependent transcription factor network. Our observation that Dicer 1 inactivation reduces Syt1 mRNA, while enhancing neurofilament message levels, suggests that Dicer 1-dependent processes contribute to the divergent expression patterns of pan-neuronal and synaptic protein genes in differentiating neurons. In addition, Dicer 1 could be required for generation or maintenance of a subpopulation of sympathetic neurons with high Syt1 and low neurofilament mRNA levels. The decrease in neuronal SCG10 mRNA levels demonstrates that distinct pan-neuronal markers are differently affected. This is supported by the observation that a TuJ1 signal persists in sympathetic neurons from mice with Dicer 1 inactivation in neural crest cells [30].

In summary, while the importance of a Phox2b-dependent transcription factor network for induction of sympathoadrenal differentiation is well established, its role in generic neuronal differentiation remains unclear. The intriguing alterations of synaptic protein and neurofilament mRNA levels upon Dicer 1 inactivation prompt the question for the role of microRNAs in this aspect of neuronal maturation.

### Neuronal marker regulation is affected by Dicer 1 during neuroendocrine differentiation

Repression of neurofilament expression is a hallmark of chromaffin cell differentiation from sympathoadrenal progenitors in rodent adrenal medulla [16,17]. However, the progenitors in mammals and birds are characterized by expression of distinct neuronal features. While mouse precursor cells in the adrenal anlage show prominent but transient NF-M and NF-L mRNA signals (this study), these cells in chick embryos lack NF-M but express SCG10 mRNA [10]. The findings indicate that the regulation of pan-neuronal genes is not well conserved in adrenal cells in the different vertebrate phyla.

In rodents, neurofilament repression during progenitor differentiation involves Dicer 1 activity. Dicer 1 inactivation in differentiated catecholaminergic cells derepresses NF-M and NF-L mRNA levels in adrenal chromaffin cells. NF-L expression is also observed in the adrenal medulla of mammalian achaete-scute homolog 1 (Mash1), also known as achaete-scute homolog 1 (Ascl1), mutant mice [17]. While these animals show increased numbers of cells with neuroblast-like ultrastructure in the embryonic adrenal medulla, such alterations are not observed in newborn animals with Dicer 1 inactivation indicating that Ascl1 and Dicer 1-dependent regulation overlap only partially.

Apart from neurofilaments, Snap25 is observed early in the mouse (this study) and rat [58] sympathoadrenal system. During embryonic development, neuronal mRNA levels become rapidly increased, while those in the adrenal medulla appear downregulated, albeit not to the same extent as neurofilaments. Decreased neuronal but maintained neuroendocrine Snap25 mRNA levels after mutational inactivation indicate Dicer 1 requirement for neuronal induction, but not neuroendocrine regulation during embryogenesis.

Taken together, Dicer 1 activity in adrenal chromaffin cells is involved in the repression of pan-neuronal but not synaptic protein mRNAs. Thus, Dicer 1 is not responsible for the generally low expression of synaptic protein genes in neuroendocrine cells during embryogenesis. Ascl1 similarly affects neurofilament expression in adrenal medulla, raising the question for the interaction with Dicer 1-dependent processes.

## Conclusions

Whereas analysis of pan-neuronal markers has long been considered sufficient to evaluate generic neuronal differentiation, functional aspects related to electrical activity and synaptic transmission receive enhanced recognition [15,59]. The regulation of physiologically relevant properties shared with other cell types, such as regulated vesicle fusion in neurons and neuroendocrine cells, poses an unresolved question of particular interest in developmental and cell biology.

Here, we demonstrate qualitatively similar but quantitatively divergent gene expression patterns for synaptic proteins in neurons and neuroendocrine cells that correlate with differences in the respective vesicle fusion mechanisms. Moreover, the developmental profiles of message regulation for synaptic protein and pan-neuronal genes differ vastly between the distinct sympathoadrenal lineages and provide evidence for cell type-specific gene regulatory programs. The consequences of Dicer 1 inactivation, in particular the partial induction of neuronal features in neuroendocrine cells, prompt the question for the mediators of Dicer 1 function and their role in cell type diversification.

## Methods

### Animals

DicerloxP mice [60] and DBHCre mice [50,52] have been described previously. DicerloxP mice were kept homozygous for the floxed Dicer allele and crossed with DBHCre mice carrying a Cre recombinase transgene under control of the DBH promoter. Plug date was counted as E0.5. Embryonic stages were confirmed according to Theiler [61].

Animals are kept at the Animal Facility of the Max Planck Institute for Brain Research, Frankfurt, Germany, according to the German Animal Welfare Act (Deutsches Tierschutzgesetz 2006, revised 2010) and applicable European Union guidelines (RL 2010/63/EU). Ethical approval was not required.

### Tissue preparation

After preparation, tissue samples were fixed with 4% paraformaldehyde (PFA) in 0.1 M sodium phosphate buffer pH 7.0 at 4°C for 24 hours. Fixative was replaced by 30% sucrose in 0.1 M sodium phosphate buffer pH 7.0 for 24 hours. Tissues were frozen in Tissue-Tek (Sakura Finetek, Alphen aan den Rijn, Netherlands) and stored at −20°C. Serial frozen sections of 12 μm were cut on a Leica CM3050 S microtome (Solms, Germany).

### Riboprobes

Riboprobes were synthesized from linearized plasmids with the DIG RNA Labeling Kit (Roche, Mannheim, Germany) according to the manufacturer’s instruction. The following mouse cRNA probes were used: DBH (987 bp; gift from J-F Brunet, Ecole Normale Supérieure (ENS), Paris, France); SCG10 (541 bp; gift from G Grenningloh, University of Lausanne, Lausanne, Switzerland); Nr5a1 (602 bp; gift from K Huber, University of Freiburg, Freiburg, Germany); NF-L (695 bp corresponding to base 418–1112 of NM_010910); NF-M (680 bp corresponding to base 2468–3147 of NM_008691); Syt1 (640 bp corresponding to base 776–1415 of NM_009306); Syt7 (749 bp corresponding to base 1127–1875 of NM_018801); Snap25 (888 bp corresponding to base 214–1106 of NM_011428); and Rab3A (682 bp corresponding to base 527–1208 of NM_00116639).

### In situ hybridization (ISH) with conventional cRNA probes

Nonradioactive ISH on cryosections was performed as described previously [62] with the following modifications: hybridization and subsequent washing was performed at 68°C, washing with maleic acid buffer was carried out 4 × 15 minutes, sections were blocked for 2 hours, and anti-digoxigenin (DIG) alkaline phosphatase conjugate was diluted 1:4,000. The color reaction was performed consistently for 18 hours using 10 μL nitro blue tetrazolium chloride (NBT)/5-bromo-4-chloro-3-indolyl phosphate (BCIP) Stock Solution (Roche) per mL buffer. ISH was performed in parallel on every tenth section of three control mice or of three control mice and three Dicer mutant mice.

Digital photography for figures and quantitative analysis was performed using a Axiophot 2 microscope (Zeiss, Oberkochen, Germany) in combination with a Spot RT3 camera (Visitron Systems, Pucheim, Germany) and adapted for brightness. MetaView software (Universal Imaging Corporation, West Chester, PA, USA) was used for quantitative analysis. For size measurement of SCG and adrenal medulla, the area was measured per section, and scaled by the number and thickness of sections to obtain the volume. As the DBH ISH signal in sympathetic ganglia and adrenal medulla is strong but restricted to catecholaminergic neurons, small intensely fluorescent (SIF) and chromaffin cells, and accurately spares the cells of the ganglion sheaths and adrenal cortex as well as blood vessels, DBH ISH provides a measure of the size of noradrenergic/adrenergic cell populations in the tissues. To analyze cells with different expression levels for the probes studied, signal intensities across the ganglion sections were grouped to reflect strong, intermediate and weak strength. The proportions of section area displaying defined ranges of signal strength were compared between genotypes to provide evidence for alterations in cell populations or mRNA levels.

### miRNA ISH

ISH was performed on 10 μm thick cryosections from E16.5 control and mutant mouse embryos. Air dried sections were fixed with 4% PFA for 10 minutes at room temperature (RT). Sections were acetylated in acetic anhydride/triethanolamine for 10 minutes at RT followed by PBS washes. Sections were permeabilized using proteinase K solution (5 μg/mL) and washed in PBS. ISH was carried out using a 5′ DIG-labeled LNA modified probe (Exiqon, Vedbaek, Denmark) complementary to the miRNA or scrambled control (3 pmol) at a temperature 20°C lower than melting temperature of the probe. Post-hybridization washes with 5 × saline sodium citrate (SSC) and 0.2 × SSC were carried out at the same temperature as that of the hybridization for 15 minutes and 1 hour, respectively. Tissue was blocked with lamb serum followed by incubation with anti-DIG alkaline phosphatase in blocking reagent (1:1,500) overnight at 4°C. NBT/BCIP was used as the substrate to detect the signal. Color reaction was stopped after 48 hours by washing in PBS. Slides were mounted with Aquatex (Boehringer, Mannheim, Germany) and analyzed with bright field microscopy (Axioplan 2, Zeiss).

### Electron microscopy

P0 control and mutant mice were perfused with 1.5% formaldehyde and 1.5% glutaraldehyde in 0.1 M PBS (pH 7.4). Adrenal glands were dissected out from the perfused animals and further fixed in the same fixative for 2 hours at RT. Fixed adrenals were washed in 0.1 M PBS for 3 × 15 minutes. Tissue was treated with 1% osmium tetroxide in 0.1 M PBS for 2 hours at RT followed by block staining in uranyl acetate. Following dehydration in a graded series of ethanol and propylene oxide, tissue was embedded in Durcupan (Fluka, St Louis, MO, USA). Ultrathin sections of 65 nm were examined with an electron microscope (Leo 906E, Zeiss) and images were captured using a 2 K CCD Camera Sharp-eye (Troendle, Moorenweis, Germany).

For quantitative analysis, ultrathin sections from six different planes, at least one cell thickness apart, were collected from both adrenals for each animal. Ten chromaffin cells from each plane were analyzed adding to 120 cells per animal. Images were analyzed to calculate numbers of chromaffin cells per unit cytoplasmic area and area of chromaffin granules using iTEM software (Olympus, Tokyo, Japan).

## Abbreviations

Ascl1: Achaete-scute homolog 1; BCIP: 5-bromo-4-chloro-3-indolyl phosphate; DBH: Dopamine β-hydroxylase; DIG: Digoxigenin; GFP: Green fluorescent protein; ISH: In situ hybridization; LDCV: Large dense-core vesicle; LNA: Locked nucleic acid; Mash1: Mammalian achaete-scute homolog 1; miRNA: MicroRNA; NBT: Nitro blue tetrazolium chloride; NF-L: Low molecular weight neurofilament protein; NF-M: Medium molecular weight neurofilament protein; Nr5a1: Nuclear receptor subfamily 5, group A, member 1; P: Postnatal day; PBS: Phosphate-buffered saline; PFA: Paraformaldehyde; Phox2b: Paired-like homeobox 2b; Rab3a: Ras-related protein Rab-3A; RT: Room temperature; SCG: Superior cervical ganglia; SCG10: Superior cervical ganglia, neural specific 10; SF1: Steroidogenic factor 1; SIF: Small intensely fluorescent; Snap25: Synaptosomal-associated protein 25; SSC: Saline sodium citrate; Syt1: Synaptotagmin I; Syt7: Synaptotagmin VII; TH: Tyrosine hydroxylase.

## Competing interests

The authors declare that they have no competing interests.

## Authors’ contributions

JS managed the mouse lines, prepared the tissues, constructed ISH probes for mRNA, and performed and analyzed ISH. PN contributed toward performing and interpreting miRNA ISHs, and ultrastructural analysis. KH contributed to the design and interpretation of the ultrastructural analysis and the miRNA ISHs. KU supervised parts of the adrenal analysis and helped to draft the manuscript. HR participated in the study design and coordination, and helped to draft the manuscript. UE developed the project, participated in the study design and coordination, and wrote the manuscript. All authors read and approved the final manuscript.
